# Diagnostic model for predicting hyperuricemia based on alterations of the gut microbiome in individuals with different serum uric acid levels

**DOI:** 10.3389/fendo.2022.925119

**Published:** 2022-09-27

**Authors:** Meiting Liang, Jingkun Liu, Wujin Chen, Yi He, Mayina Kahaer, Rui Li, Tingting Tian, Yezhou Liu, Bing Bai, Yuena Cui, Shanshan Yang, Wenjuan Xiong, Yan Ma, Bei Zhang, Yuping Sun

**Affiliations:** ^1^ Departent of Microbiology, School of Basic Medical Sciences, Xinjiang Medical University, Urumqi, China; ^2^ Department of Pathology, School of Basic Medical Sciences, XinJiang Second Medical College, Karamay, China; ^3^ Department of Oncology, The First Affiliated Hospital of XinJiang Medical University, Urumqi, China; ^4^ Department of Morphological Center, School of Basic Medical Sciences, Xinjiang Medical University, Urumqi, China; ^5^ Department of Human Parasitology, School of Basic Medical Sciences, Xinjiang Medical University, Urumqi, China; ^6^ Department of Clinical Laboratory, Xinjiang Medical University Affiliated Fifth People’s Hospital, Urumqi, China; ^7^ Department of Clinical Laboratory, Xinjiang Medical University Affiliated Second People’s Hospital, Urumqi, China; ^8^ Key Laboratory of Xinjiang Uygur Autonomous Region, Laboratory of Molecular Biology of Endemic Diseases, Urumqi, China

**Keywords:** gut microbiome, uric acid, hyperuricemia, diagnostic model, nomogram

## Abstract

**Background:**

We aimed to assess the differences in the gut microbiome among participants with different uric acid levels (hyperuricemia [HUA] patients, low serum uric acid [LSU] patients, and controls with normal levels) and to develop a model to predict HUA based on microbial biomarkers.

**Methods:**

We sequenced the V3-V4 variable region of the 16S rDNA gene in 168 fecal samples from HUA patients (n=50), LSU patients (n=61), and controls (n=57). We then analyzed the differences in the gut microbiome between these groups. To identify gut microbial biomarkers, the 107 HUA patients and controls were randomly divided (2:1) into development and validation groups and 10-fold cross-validation of a random forest model was performed. We then established three diagnostic models: a clinical model, microbial biomarker model, and combined model.

**Results:**

The gut microbial α diversity, in terms of the Shannon and Simpson indices, was decreased in LSU and HUA patients compared to controls, but only the decreases in the HUA group were significant (P=0.0029 and P=0.013, respectively). The phylum *Proteobacteria* (*P*<0.001) and genus *Bacteroides* (*P*=0.02) were significantly increased in HUA patients compared to controls, while the genus *Ruminococcaceae_Ruminococcus* was decreased (*P*=0.02). Twelve microbial biomarkers were identified. The area under the curve (AUC) for these biomarkers in the development group was 84.9% (*P*<0.001). Notably, an AUC of 89.1% (*P*<0.001) was achieved by combining the microbial biomarkers and clinical factors.

**Conclusions:**

The combined model is a reliable tool for predicting HUA and could be used to assist in the clinical evaluation of patients and prevention of HUA.

## Introduction

Uric acid is the end product of purine metabolism. The body excretes uric acid in two main ways: approximately 70% is excreted in urine *via* the kidneys and approximately 30% is transported by intestinal epithelial cells to the lumen for direct excretion or decomposition by gut microbes (1). Generally, uric acid metabolism is in a dynamic balance, but with changes in lifestyle, along with the influence of genetic factors, serum uric acid levels can become too high or too low.

Hyperuricemia (HUA) is diagnosed if the fasting blood uric acid level is >7 mg/dL in men and >6 mg/dL in women (2). Research has confirmed that HUA can cause kidney, cardiovascular, liver, and soft tissue damage (3). The main mechanism involves activating the oxidative stress response and promoting the release of inflammatory factors (4). Accordingly, the incidences of dyslipidemia, type 2 diabetes, and cardiovascular disease are higher in HUA patients than in healthy subjects (5–8). HUA is also a prerequisite for gout; continuously elevated serum uric acid can cause sodium urate crystal precipitation due to oversaturated urate, leading to gout. The cumulative incidence of gout increases with serum uric acid levels (9, 10).

Furthermore, low serum uric acid (LSU) is often overlooked. Some studies have shown that LSU (male <3.5 mg/dl and female <2.5 mg/dl) is associated with all-cause mortality and gender-specific cardiovascular mortality (11), but the overall relationship between cardiovascular disease risk and mortality are not clear. The harm of LSU may be related to the antioxidant properties of uric acid (11, 12).

The gut microbiota is a huge microbial population that colonizes the intestine and is symbiotic with the host. It plays an important role in functional metabolism and immune regulation. Many studies have found that it is related to uric acid metabolism disorders. Shao et al. (13) found that the diversity of the intestinal microbiota decreased in patients with gout, with increases in the opportunistic pathogens *Bacteroides* spp., *Porphyromonadaceae*, *Rhodococcus*, *Erysipelatoclostridium* spp., and *Anaerolineaceae* and decreases in *Lachnospiraceae* and *Ruminococcaceae*. In contrast, other gut microbes are capable of reducing intestinal uric acid production and/or increasing uric acid excretion. For example, *Lactobacillus gasseri* PA3 reduces exogenous purine absorption, including inosine 5-phosphate (IMP), inosine, hypoxanthine, guanosine 5-phosphate (GMP), and guanine nucleoside (14). Furthermore, *Lactobacillus* DM9218 isolated from pickles can degrade inosine and guanosine (two critical intermediates in purine metabolism), reduce the production of uric acid precursors, and effectively reduce serum uric acid levels (15). In summary, in the intestinal tract, which is an important site for uric acid excretion, the microbiome plays an indispensable role. It may be feasible to use the gut microbiome as a predictor of uric acid metabolic disorders.

The utility of using traditional clinical factors for predicting HUA, to better evaluate HUA occurrence and development, is limited. In current clinical practice, the serum uric acid level is still used to diagnose HUA, but the occurrence and development of HUA cannot be easily pre-empted. Therefore, it is important to establish a new diagnostic model involving both traditional clinical factors and gut microbial biomarkers. Controlling daily purine intake is an effective means to prevent HUA, but this is often disregarded at present; if cases of HUA could be effectively predicted, controlling daily purine intake may be a highly useful intervention.

To establish a new diagnostic model, we aimed to analyze the gut microbiota in participants with different uric acid levels by high-throughput sequencing. Using gut microbial biomarkers and clinical factors, we developed a novel diagnostic model for HUA and used decision curve analysis (DCA) to assess its clinical value.

## Methods

### Study participants

The study participants were aged 20–60 years old and were recruited from Balikun County Hospital, Xinjiang Province of China, from October 2019 to August 2021. All participants underwent physical examination in Balikun County Hospital. Height, weight, body mass index (BMI), blood pressure (BP), genetic and disease history, and drug use history were recorded.

The study cohort was divided into the following three groups according to mean serum uric acid levels (measured on 2 days): LSU group (males: <3.5 mg/dL; females: <2.5 mg/dL; n=61) (11), control group (n=57), and HUA group (males: >7 mg/dL; females: >6 mg/dL; n=50) (2).The exclusion criteria were gastrointestinal diseases, tumors, hematological diseases, severe liver or kidney dysfunction, and the use of antibiotics or microecological regulators that affect the gut microbiota within 1 month of the study.

The study was approved by the Ethics Committee of the First Affiliated Hospital of Xinjiang Medical University (Xinjiang, China). The sampling and other methods were carried out according to the approved guidelines. Each participant was informed about the study before study commencement, and they signed an informed consent form.

### Fecal sample collection, DNA extraction, and sequencing

We provided each participant with a sterile container for stool sample collection and training on sample collection. After stool sample collection, 0.2 g fresh fecal sample from each participant was quickly placed into five sterile centrifuge tubes and rapidly transported to the laboratory for freezing at -80°C. The bacterial DNA was extracted from the samples using the bead-beating method. The quality of DNA was assessed using 0.8% agarose gel electrophoresis (16). The DNA was used as the template to amplify the V3-V4 region of the bacterial 16S rDNA gene. The DNA extraction and Illumina NovaSeq sequencing were performed by Shanghai Pisenno Biotechnology Co., Ltd. (Shanghai, China; http://www.personalbio.cn).

### Gut microbiome analyses

Operational taxonomic unit (OTU) clustering analysis was performed on all sequences. The Ribosomal Database Project (RDP) Bayesian classifier was used to classify the representative OTU sequences at the 97% similarity level. The community composition of each sample was assessed according to various classification levels (phylum, class, order, family, and genus), and the community composition was statistically compared between groups at each classification level. We used the heatmap R package to present clustering results at phylum and genus levels in heatmaps.

α diversity analyses were used to assess the abundance and diversity (Shannon and Simpson indices) of microbial communities in the three uric acid-related groups. Using principal coordinates analysis (PCoA) of UniFrac distances and nonparametric analysis of similarities (ANOSIM), we assessed the similarity between the three uric acid-related groups. Additionally, we used linear discriminant analysis (LDA) effect size (LEfSe) to determine the specific gut microbes related to HUA.

### Laboratory assessment

Peripheral venous blood samples of all participants were obtained after 12 h of fasting. Laboratory data, including serum biochemical indexes, liver function indexes, renal function indexes, and lipid results, were examined in the Balikun County Hospital as described previously.

### Statistical analysis

We established three diagnostic models: a clinical model, microbial biomarker model, and combined model. To establish the clinical model, we used univariate logistic regression analyses to identify significant clinical factors for predicting HUA followed by a receiver operating characteristic (ROC) curve analysis of each factor to select the most valuable factors for predicting HUA, based on the area under the ROC curve (AUC) values. Next, using the significant variables with AUC value >0.5, we used least absolute shrinkage and selection operator (LASSO) regression to determine the independent risk factors for HUA. The available clinical factors included blood pressure, blood urea nitrogen (BUN), creatinine, blood glucose, alanine aminotransferase, aspartate aminotransferase, triglyceride (TG), total cholesterol (TC), high-density lipoprotein cholesterol (HDL-C), and low-density lipoprotein cholesterol (LDL-C).

To establish the microbial biomarker model, we used 10-fold cross-validation of a random forest model to ensure the reliability of the gut biomarkers. We randomly divided (2:1 ratio) the 107 volunteers in the HUA and control groups into the development and validation groups, and the former was used to construct the microbial biomarker model, while the latter was used to assess model reliability. The probability of disease (POD) index in the HUA and control samples in the development and validation groups was used to determine the diagnostic value of the gut microbial biomarkers. It was defined as the ratio between the number of decision trees that predicted “HUA” and the total number of sampling trees (in the development or validation group, n_votes_/n_trees_).

All variables from the other two models were included in the combined model. The combined model was visualized by constructing a nomogram using the rms R package. DCA was used to assess the clinical value of the combined model. Additionally, calibration curves comparing the model predictions and actual observations were used for graphical evaluation.

Statistical analysis was performed using SPSS software (version 21.0; IBM Corp., Armonk, NY, USA) and R software (version 4.0.4; R Foundation for Statistical Computing, Vienna, Austria). The normally and non-normally distributed continuous variables are expressed as mean ± standard deviation (mean ± SD) and median, respectively. The categorical variables are presented as percentages. Regarding normally distributed continuous variables, the three uric acid-related groups were compared using a one-way analysis of variance (ANOVA). Differences between the HUA and control groups were determined using *t* tests for normally distributed continuous variables and Wilcoxon rank-sum test for non-normally distributed continuous variables. Regarding categorical variables, groups were compared using the Chi-square test. P<0.05 was considered statistically significant.

## Results

### Participant characteristics

**Figure 1:**
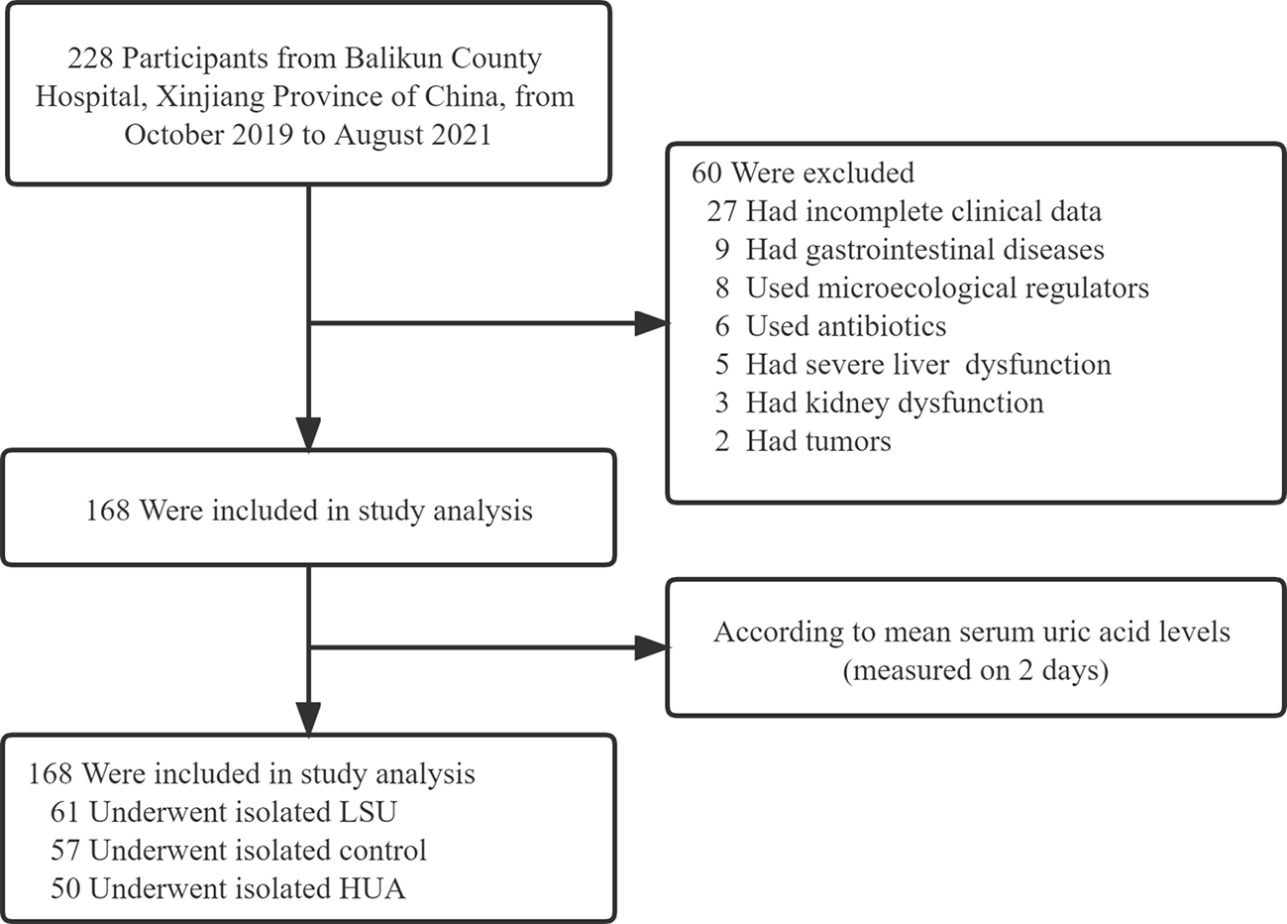
Flow chart of the data screening.

The study cohort comprised 168 Chinese adults, and it was divided into the LSU, control, and HUA groups (**Figure 1**). There were no significant differences in age, gender, or body mass index among the three groups, but there were significant differences in uric acid, BUN, creatinine, blood glucose, alanine aminotransferase, TG, HDL-C, and LDL-C (**Table 1**). The mean BUN, creatinine, blood glucose, TG, and LDL-C were higher in the HUA group and lower in the LSU group relative to the control group. These results show that the level of uric acid is associated with glucose and lipid metabolism, and HUA patients may be more prone to glucose and lipid metabolism disorders and related diseases. Therefore, predicting HUA occurrence and development should involve these clinical biochemical factors, to ensure a comprehensive evaluation.

**Table 1:** Participant characteristics.

Variable	Low serum uric acid (LSU) group	Control group	Hyperuricemia group	*X^2^* or *F*	*P* value
Age, years	46.25±9.83	47.19±9.64	49.18±8.21	1.395	0.251
Sex, male, n (%)	40(65.6)	36(63.2)	33 (66)	5.587	0.061
Body mass index	25.70±4.31	26.36±3.66	27.13±3.62	1.857	0.159
SBP (mmHg)	125.93±20.68	131.26±19.94	127.46±16.49	1.179	0.31
DBP (mmHg)	76.92±10.13	78.30±11.14	77.34±13.32	0.22	0.802
Uric acid (µmol/l)	162.13±30.62	274.69±48.93	451.43±65.81	147.1	<0.001
Blood urea nitrogen (mmol/l)	3.86±1.17	4.29±1.15	5.58±1.73	19.793	<0.001
Creatinine (µmol/l)	64.35±17.27	72.99±13.66	73.20±15.60	6.082	0.003
Blood glucose (mmol/l)	4.76±0.60	5.12±0.62	5.82±2.26	11.531	0.003
Alanine aminotransferase (U/L)	19.16±9.59	24.93±11.06	20.8±9.85	4.962	0.008
Aspartate transaminase (U/L)	25.95±10.75	23.74±10.32	23.36±11.16	0.98	0.377
Triglyceride (mmol/l)	1.07±0.29	1.18±0.74	1.94±0.94	37.979	<0.001
Total cholesterol (mmol /l)	3.69±1.22	3.98±1.62	4.13±1.08	1.593	0.207
HDL-C (mmol/l)	1.40±0.29	1.56±0.37	1.13±0.34	22.291	<0.001
LDL-C (mmol/l)	2.25±0.6	2.64±0.58	2.65±0.9	10.652	0.005

SBP, systolic blood pressure; DBP, diastolic blood pressure; HDL-C, high-density lipoprotein cholesterol; LDL-C, low-density lipoprotein cholesterol.

### Alterations in the gut microbiome by serum uric acid level

**Figure 2:**
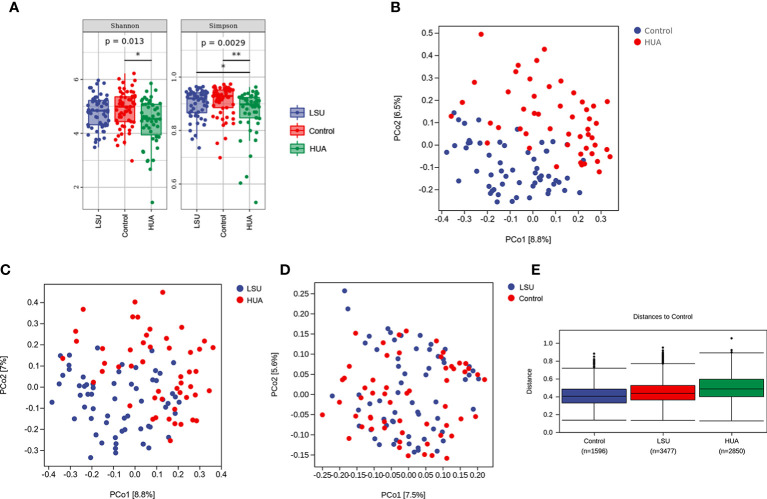
Serum uric acid level was associated with changes in the gut microbiota. **(A)** α diversity analyses based on Simpson and Shannon indices. Simpson and Shannon indices in the low serum uric acid (LSU) and hyperuricemia (HUA) groups decreased compared to the control group, but only the decreases in the HUA group were significant (P=0.0029 and P=0.013, respectively). **(B–D)** β diversity was assessed using principal coordinates analysis (PCoA) of unweighted UniFrac distances, which reflected the dispersion degree of the sample point distribution. There were large differences in the composition of the gut microbiota between the HUA and control groups. **(E)** Nonparametric analysis of similarities (ANOSIM) among the three groups showing that the LSU and HUA groups were far away from the control group. The control group box plot indicates the sample differences within the control group. The other two box plots indicate the distance from the HUA or LSU groups to the control group. The number corresponding to x-axis represents the number of comparisons between samples, and the y-axis represents the distance.

The Simpson and Shannon indices (representing gut microbial α diversity) were significantly lower in the LSU and HUA groups than in the control group, but only the decreases in the HUA group were significant (P=0.0029 and P=0.013, respectively, **Figure 2A**), indicating that alterations in serum uric acid level were related to lower gut microbial α diversity.

In addition, based on principal coordinates analysis (PCoA) of UniFrac distances, there were significant separations between the HUA group and the other two groups (**Figures 2B, C**), but there was no significant separation between the LSU and control groups (**Figure 2D**). These results showed that the gut microbial community structure in the HUA group was significantly altered (**Figure 2B**). Furthermore, nonparametric analysis of similarities (ANOSIM) also showed that the distribution and composition of gut microbial communities differed with uric acid level (**Figure 2E** and **Table S1**).

### Gut microbes in HUA

**Figure 3:**
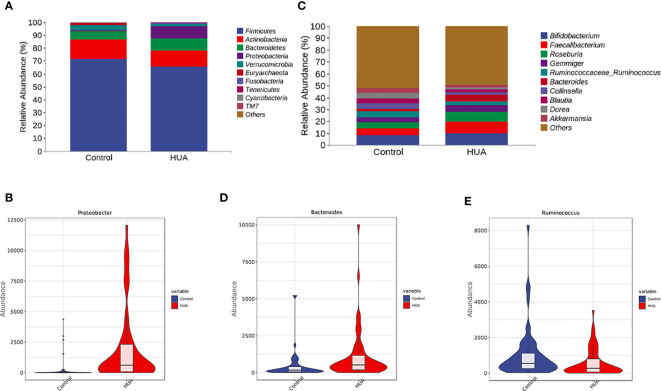
Differences in gut microbiota between the HUA and control groups. **(A)** Dominant phyla in HUA and control groups and **(B)** difference in Proteobacteria. **(C)** Dominant genera in HUA and control groups, and differences in **(D)** Bacteroides (P=0.02) and **(E)** *Ruminococcaceae_Ruminococcus* (P=0.04).

We presented the clustering results at phylum and genus levels in heatmaps. *Firmicutes*, *Actinobacteria*, *Bacteroidetes*, *Proteobacteria*, and *Verrucomicrobia* were the dominant phyla in the HUA group (**Figure 3A**). *Proteobacteria* was significantly increased in the HUA group compared to the control group (P<0.001, **Figure 3B**). At the genus level (**Figure 3C**), *Bacteroides* was higher in the HUA group than the control group (*P*=0.02, **Figure 3D**), while *Ruminococcaceae_Ruminococcus* was lower (*P*=0.04, **Figure 3E**).

**Figure 4:**
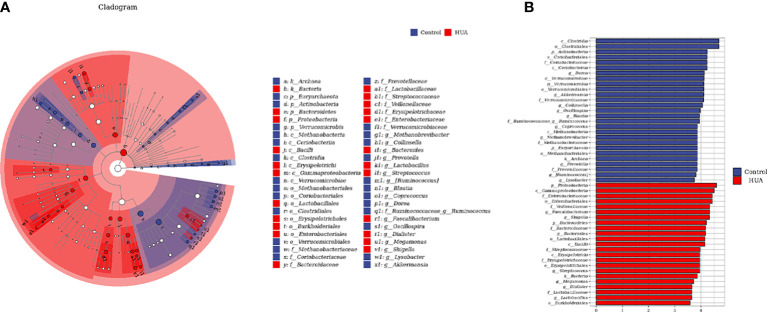
Linear discriminant analysis (LDA) effect size (LEfSe) analysis indicating the most differential genera between the HUA and control groups. **(A)** Cladogram. The brightness of each point is directly proportional to its effect. **(B)** Histogram. Blue and red represent control and HUA samples, respectively.

To determine the specific gut microbes related to HUA, we used linear discriminant analysis (LDA) effect size (LEfSe) to screen 186 gut microbial species. **Figures 4A, B** show the top 50. The phylum *Bacteroidetes* and its derivatives (*Bacteroidia*, *Bacteroidaceae*, and *Bacteroides*), the phylum *Proteobacteria* and its derivative (*Gammaproteobacteria*), the order *Enterobacteriales* and its derivatives (*Enterobacteriaceae* and *Shigella*), the class *Erysipelotrichi* and its derivatives (*Erysipelotrichales* and *Erysipelotrichaceae*), the family *Streptococcaceae* and its derivative (*Streptococcus*), and the order *Lactobacillales* and its derivatives (*Lactobacillaceae* and *Lactobacillus*) were higher in the HUA patients. Conversely, the phylum *Verrucomicrobia* and its derivatives (*Verrucomicrobiae*, *Verrucomicrobiales*, *Verrucomicrobiaceae*, and *Akkermansia*), the phylum *Actinobacteria* and its derivatives (*Coriobacteriia*, *Coriobacteriales*, *Coriobacteriaceae*, and *Collinsella*), and the family *Ruminococcaceae* and its derivative (*Ruminococcus*) were lower in the HUA patients.

### Microbial biomarker model for diagnosing HUA

**Figure 5:**
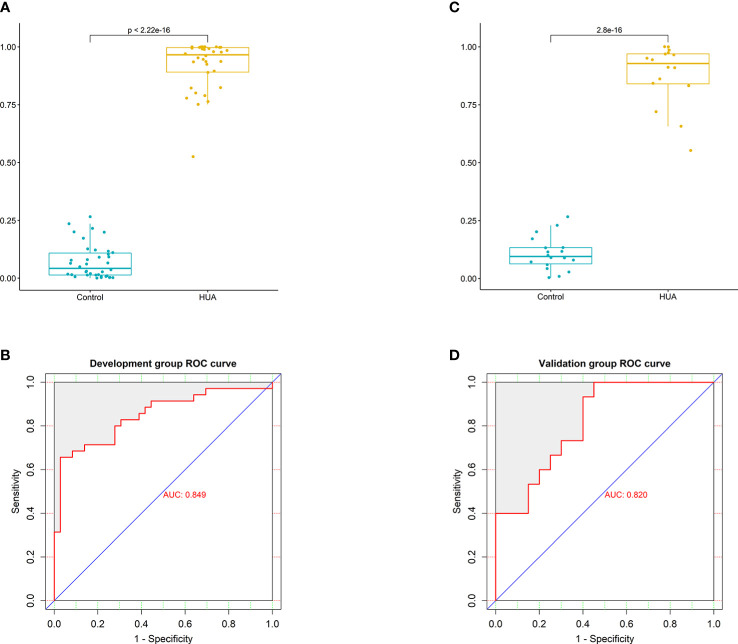
Performance of the microbial biomarker model for diagnosing HUA. **(A)** Comparison of the probability of disease (POD) index between the HUA and control groups and **(B)** ROC curve (with AUC) in the development group. **(C)** Comparison of the POD index between the HUA and control groups and **(D)** ROC curve (with AUC) in the validation group.

To develop a HUA diagnostic model using gut microbial biomarkers, we randomly divided (2:1 ratio) 107 participants (from the HUA and control groups) into the development and validation groups. Subsequently, we performed a 10-fold cross-validation of the random forest model in the development group, and 12 gut microbial biomarkers were identified as the optimum parameter set (**Table S2**). The POD index in HUA and control samples in the development and validation groups was used to determine the diagnostic value of the 12 gut microbial biomarkers. The POD index was significantly increased in the HUA samples compared to the control samples in both groups (both P<0.001, **Figures 5A, C**). The POD index achieved an AUC value of 84.9% and 82% for distinguishing between the HUA patients and controls in the development and validation group, respectively (**Figures 5B, D**). To evaluate the discriminatory ability of the ROC curve, we computed the AUC with a 95% CI by using 500 bootstrap resamplings. Sensitivity, specificity, positive predictive value (PPV), negative predictive value (NPV), positive likelihood ratio (PLR), and negative likelihood ratio (NLR) of the stepwise model are also presented in **Table 2** (which shows the clinical model and the combined model, similarly hereinafter). This suggested that the microbial biomarker model has a high diagnostic value for predicting HUA.

**Table 2:** Prediction Performance of the Three Models.

	Microbial Marker Model		Clinical Model		combined model		
	Development group	Validation group	Development group	Validation group	Development group		Validation group
AUC (95%CI)	0.849 (0.752- 0.945)	0.820 (0.688-0.988)	0.810 (0.726-0.890)	0.801(0.635-0.967)	0.891 (0.817-0.966)	0.862 (0.736-0.988)	
Cutoff value	58.4%	49.6%	56.4%	48.7%	40.7%		67.6%
Sensitivity, %	91.7%	71.4%	69.4%	78.6%	80.6%		78.9%
Specificity, %	79.4%	95.5%	83.3%	71.4%	85.4%		81.2%
PPV, %	82.5%	90.9%	80.6%	64.7%	80.6%		83.3%
NPV, %	90.0%	84.0%	73.2%	83.3%	85.4%		76.5%

AUC, area under curve; PPV, positive predictive value; NPV, negative predictive value.

### Combined model vs clinical and microbial marker models for diagnosing HUA

The clinical model was constructed using three clinical factors (BUN, TG, and HDL-C), and these factors were then also used in the combined model. These three clinical factors were selected using univariate regression and ROC curves (with AUCs), which identified 11 predictive clinical factors (**Table 3**), followed by least absolute shrinkage and selection operator (LASSO) regression of the 10 variables with AUC >0.5.

**Table 3:** Candidate Variables for Clinical Model Development.

variables	AUC	*P* values	95% CI
SBP	0.6003	0.07226232	0.4665-0.7341
DBP	0.5517	0.2267171	0.4171-0.6863
BUN	0.7168	0.000790465	0.599-0.8347
Cr	0.5637	0.1778288	0.4284-0.6989
glucose	0.5498	0.2355044	0.4105-0.689
ALT	0.6385	0.02189498	0.5096-0.7674
AST	0.5475	0.2460171	0.4126-0.6823
TG	0.799	6.48E-06	0.6824-0.9156
TC	0.5544	0.2152263	0.4198-0.689
HDL	0.8098	3.14E-06	0.7117-0.9079
LDL	0.4996	0.5044931	0.3576-0.6416

**Figure 6:**
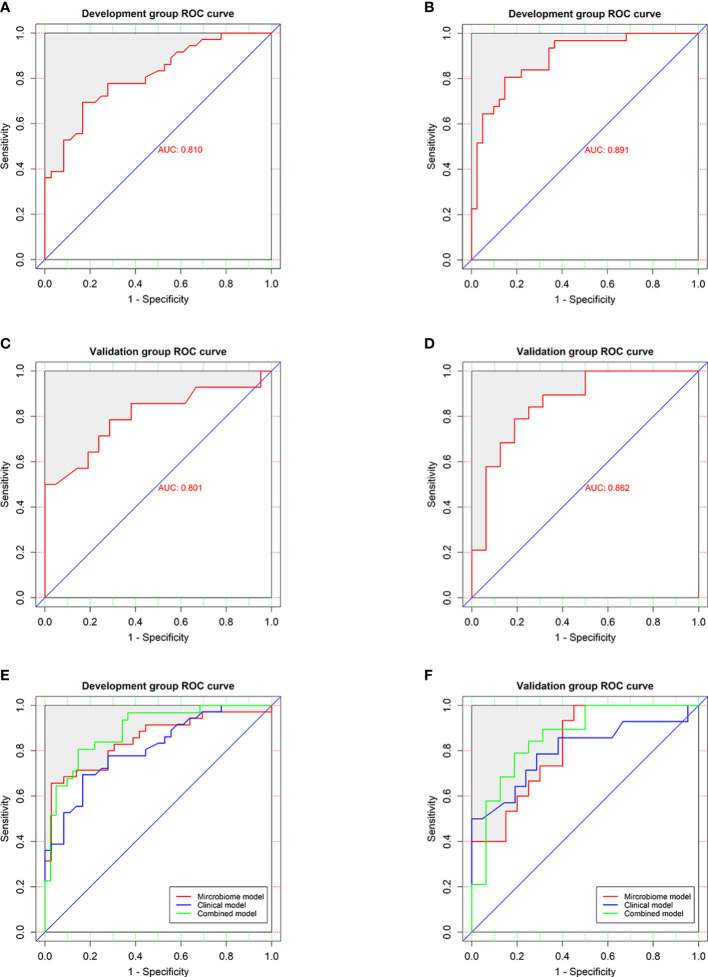
Performances of the clinical and combined models for diagnosing HUA. ROC curves (with AUCs) for the **(A)** clinical and **(B)** combined models in the development group were similar to those for the **(C)** clinical and **(D)** combined models in the validation group. ROC curves (with AUCs) of clinical model, microbial biomarker model, and combined model in the **(E)** development and **(F)** validation groups showing that the combined model was superior to the others models in both groups. The diagonal line (45°) indicates the performance of a diagnostic test that is no better than chance.

In the development group, the combined model (**Figures 6A, B**) was superior to the clinical model (AUC: 89.1 vs 81%, P<0.001) and the microbial biomarker model (AUC: 89.1 vs 84.9%, P<0.001), while the clinical and microbial biomarker models did not significantly differ. Similarly, in the validation group, the combined model (**Figures 6C, D**) was superior to the clinical model (AUC: 86.2 vs 80.1%, P<0.001) and the microbial biomarker model (AUC: 86.2 vs 82%, P<0.001), while the clinical and microbial biomarker models did not significantly differ. In summary, the ROC curves of the three models in the development and validation groups indicated that the prediction results of the combined model were superior to those of the microbial biomarker and clinical models (**Figures 6E, F**).

### Combined model and nomogram for predicting HUA

**Figure 7:**
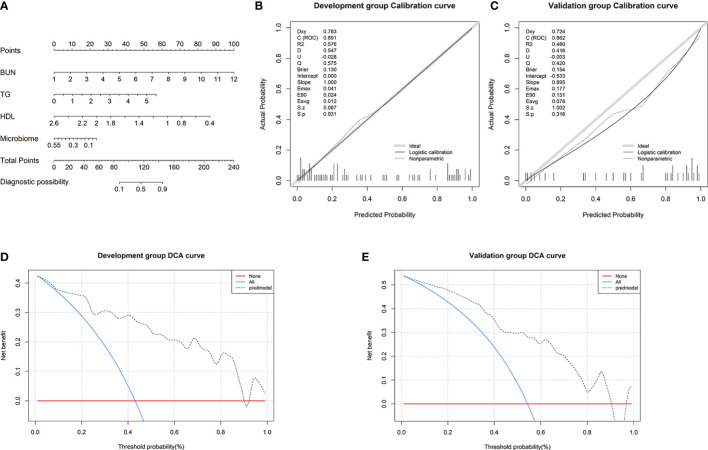
Nomogram and its performance. **(A)** To use the nomogram, draw a vertical line from the risk factor to the “Points” axis to determine the score of each risk factor in the nomogram. The total score is the sum of the scores of all risk factors. To estimate the probability of hyperuricemia (HUA), draw a vertical line from the “Total points” axis to the “Probability of HUA” axis. Calibration curves in the **(B)** development and **(C)** validation groups showing that the combined model prediction curve and actual observation curve are very close, indicating good calibration in the both groups. Decision curve analysis **(DCA)** in the **(E)** development and **(F)** validation groups. The red horizontal line and the blue diagonal line are two extreme states: intervention for no patients and intervention for all patients, respectively. The gray dotted line indicates the net benefit for the patients based on prediction using the combined model.

To visualize the prediction of HUA using the combined model, we established a nomogram based on the combined model (**Figure 7A**). Based on the risk factors on the left side of the nomogram, it can be seen that the combined model involves four risk factors. The diagnostic index (DI) of HUA can be calculated according to the following formula for the combined model: 116.92062 + 9.09091×BUN (mmol/l) + 10.31362 × TG (mmol/l) - 39.40222 × HDL (mmol/l) - 42.84683 × microbiome.

Subsequently, we found that the results of the Hosmer–Lemeshow goodness-of-fit tests for the combined model were *P*=0.931 and *P*=0.316 in the development and validation groups, respectively (**Figures 7B, C**). This indicates that there was no significant deviation between the observed and predicted probabilities. Thus, the nomogram based on the combined model (involving microbial biomarkers and clinical factors) has a high diagnostic ability regarding HUA. To further determine the clinical value of the combined model, we used DCA to evaluate the model. In both the development and validation groups, the curve for the combined model (gray dotted line) was far above the two extreme curves (intervention for none and intervention for all: red and blue lines, respectively), indicating that the combined model provides high net clinical benefit for HUA patients (**Figures 7D, E**).

## Discussion

In this study, the composition and distribution of gut microbial communities in individuals with different uric acid levels were distinct. However, in current clinical practice, the serum uric acid level is still used to diagnose HUA, and the occurrence and development of HUA cannot be easily pre-empted. Therefore, we successfully developed a diagnostic model for HUA based on gut microbial biomarkers and clinical factors for the first time, which has clinical value according to the DCA evaluation.

More and more studies have shown that the gut microbiome is closely related to a variety of metabolic diseases, such as diabetes (16, 17), hypertension (18, 19), coronary heart disease (20, 21), hyperlipidemia (22), and gout (23, 24). Guo et al. (23) found that 17 bacterial genera (including *Bacteroides*, *Holdemania*, and *Anaerotruncus*) were enriched in gout patients, while *Faecalibacterium*, *Coprococcus*, and *Ruminococcus* were decreased. We used high-throughput sequencing of the V3-V4 region of the 16S rDNA gene to characterize the microbiome in 168 fecal samples. The α diversity decreased in LSU and HUA patients compared to controls (**Figure 2A**; **Table 3**).

Additionally, we found that certain opportunistic pathogens were enriched in HUA, such as the phylum *Bacteroidetes* (and its genera *Bacteroides*), genus *Shigella*, family *Erysipelotrichaceae*, and genus *Streptococcus* (**Figures 3D**, **4A**). *Bacteroides* enrichment has been reported to be associated with intestinal inflammation, such as inflammatory bowel disease (IBD), and autoimmune diseases, such as systemic lupus erythematosus (25), rheumatoid arthritis (26), and type I diabetes (27, 28). Certain *Bacteroides* species are considered biomarkers of IBD and their associated OmpW proteins can be used as targets for developing IBD immunotherapy (29). *Shigella* and *Erysipelotrichaceae* are considered opportunistic pathogens in the human gut and are significantly increased in Crohn’s disease (30, 31). *Erysipelotrichaceae* can produce phenyl sulfate, which impairs glomerular function and is highly associated with diabetic nephropathy (32). Lastly, *Streptococcus* is a common type of pyogenic coccus that can cause various types of pyogenic inflammation related to intestinal inflammatory responses and intestinal mucosal damage, and it can affect intestinal uric acid excretion (33, 34).

In our HUA patients, the genera *Ruminococcus*, *Coprococcus*, and *Blautia*, which produce short-chain fatty acids (SCFAs), were significantly reduced (**Figures 4A, B**). SCFAs are bacterial metabolites that can promote health by regulating intestinal immune function and maintaining the intestinal mucosal barrier (35–37). The decreased *Ruminococcus*, *Coprococcus*, and *Blautia* in HUA may decrease SCFAs in the intestine, increasing the risk of HUA. *Ruminococcus* and *Coprococcus* are mainly associated with butyrate production (24). Butyrate, a major SCFA, is mainly produced by bacterial fermentation of dietary fiber in the gut. It is the main energy supply for intestinal epithelial cells (via fatty acid oxidation) and regulates intestinal health, inhibits inflammation, and has antioxidant and anticancer effects (38, 39). As mentioned, *Ruminococcus* was decreased in our HUA patients (**Figure 3E**). Chu et al. (24) also found that butyrate-producing bacteria were significantly reduced in gout patients. Additionally, long-term alcohol intake greatly decreases *Ruminococcus* in the gut, thereby affecting butyrate production and increasing the risk of steatohepatitis and liver injury; decreased *Ruminococcus* is also associated with lipid metabolism disorders, and dyslipidemia is an important risk factor for HUA (40). Furthermore, Wan et al. (41) found that a low-fat diet was associated with increased *Blautia*, and the butyrate in the feces of the high-fat diet group was significantly lower than in other groups. This indicates that a long-term high-fat diet had harmful effects on the gut microbiome, increasing the risk of inflammation and chronic diseases.

Thus, it could be seen that there were significant differences in the distribution of intestinal flora between HUA and healthy people in our research. In particular, gut microbes that produced SCFAs were significantly reduced in HUA. SCFAs are involved in energy metabolism (42); they can provide energy for intestinal epithelial cells and participate in the excretion of uric acid. The decrease in gut uric acid excretion increases the burden on the kidneys (43), and then affects the level of serum uric acid.

Subsequently, we used a random forest model to identify 12 gut microbial biomarkers as the optimal parameter set for predicting HUA. We then used the POD index of the 12 microbial biomarkers to predict HUA. This index had strong predictive ability in both the development and validation groups (AUC=84.9% and 82%, respectively; *P*<0.001). These results suggested that the microbial biomarker model might be useful for the early diagnosis of HUA.

In recent years, clinical factors have often been used to construct diagnostic prediction models. However, the model constructed using only clinical factors had only moderate predictive ability for HUA, and a combined model based on clinical factors and gut microbial biomarkers had not been previously developed for HUA. Therefore, we not only constructed a clinical model and a microbial biomarker model, but also used the factors in these two models to construct a combined model. The predictive ability of the combined model was significantly better than that of the other two models. To visualize the combined model, we constructed a nomogram that indicated the predictive ability of each included variable. More importantly, the predictive ability of the nomogram was assessed in both the development and validation groups. The results indicated that the combined model had good accuracy (based on the ROC curve), discrimination (based on the decision curve analysis), and calibration results (based on calibration curves) for predicting HUA.

There are several limitations to this study. First, although we developed a combined model that involved gut microbial biomarkers, the specific functions of these biomarkers remain unclear. Second, we only used 16S rDNA sequencing of DNA from stool samples, without assessing fecal metabolites or blood samples. Third, the sample size was relatively small, and external validation was not conducted. Multicenter studies involving different regions/countries are needed to verify the combination model.

In conclusion, we first found that there were distinct gut microbiomes among participants with different serum uric acid levels. The gut microbial diversity decreased in LSU and HUA patients compared to controls, especially in HUA patients. Therefore, we developed a diagnostic model by combining clinical factors and gut microbial biomarkers that has practical clinical value for predicting HUA.

## Data availability statement

The original contributions presented in the study are publicly available. This data can be found here: NCBI repository, accession number PRJNA869365.

## Ethics statement

The studies involving human participants were reviewed and approved by The Medical Ethics Committee of the First Affiliated Hospital of Xinjiang Medical University(No. K202105-08). The patients/Participants provided their written informed consent to participate in this study.

## Author contributions

(I) Conception and design: ML, WC, YS. (II) Administrative support: YS, MK,YC, JL. (III) Provision of study materials or patients: YH, SY, YM. (IV) Collection and assembly of data: ML, TT, WX. (V) Data analysis and interpretation: WC, RL, BZ. (VI) Manuscript writing: all authors. (VII) Final approval of manuscript: all authors. All authors contributed to the article and approved the submitted version.

## Funding

This study was funded by the National Natural Science Foundation of China (No.81960169 and 81760169), the Natural Science Foundation of the Xinjiang Uygur Autonomous Region (No.2019D01C219 and 2021D01C275), the Project of Scientific Research Program for Universities in Xinjiang Uygur Autonomous Region (No.XJEDU2021Y054), the Postgraduate Research Innovation Project in Xinjiang Uygur Autonomous Region (No.XJ2021G228) the Science and Technology Plan Project in Xinjiang Karamay City (20212022hjcxrc0060).

## Acknowledgments

The authors are grateful to all the participants.

## Conflict of interest

The authors declare that the research was conducted in the absence of any commercial or financial relationships that could be construed as a potential conflict of interest.

## Publisher’s note

All claims expressed in this article are solely those of the authors and do not necessarily represent those of their affiliated organizations, or those of the publisher, the editors and the reviewers. Any product that may be evaluated in this article, or claim that may be made by its manufacturer, is not guaranteed or endorsed by the publisher.
