# Supplementary material for: Diagnostic model for predicting hyperuricemia based on alterations of the gut microbiome in individuals with different serum uric acid levels
Source: Front Endocrinol (Lausanne). 2022 Sep 27;13:925119. doi: 10.3389/fendo.2022.925119 (PMC9553226; doi:10.3389/fendo.2022.925119)
Supplement: Supplementary file 1 [file Table_1.pdf]

Table S1. Analysis of similarities (ANOSIM)

Group 1	Group 2	Sample size	Permutations	R	*P value*	Q value
All	-	168	999	0.115494	0.001	-
LSU	Control	118	999	0.032726	0.009	0.009
LSU	HUA	111	999	0.144347	0.001	0.0015
Control	HUA	107	999	0.18611	0.001	0.0015

Table S2. 12 gut microbial biomarkers identified by random forest model

Microbial biomarker	H.57 (mean±SD)	N.50 (mean±SD)	*P value*
Collinsella	0.046282±0.055388	0.022021±0.053160	0.023
Slackia	0.005405±0.007681	0.000960±0.003720	<0.001
unidentified_Coriobacteriaceae	0.009178±0.010915	0.001045±0.002732	<0.001
unidentified_S24_7	0.001264±0.002330	0.001470±0.004880	0.776
Odoribacter	0.000378±0.000613	0.000143±0.000363	0.02
unidentified_Christensenellaceae	0.007484±0.011112	0.000582±0.001618	<0.001
unidentified_Ruminococcaceae	0.079451±0.057019	0.021687±0.049592	<0.001
unidentified_Mogibacteriaceae.	0.002051±0.001896	0.000770±0.001131	<0.001
***unidentified_Clostridiales*	0.075592±0.076620	0.011587±0.021034	<0.001
Cupriavidus	0.000000±0.000000	0.001420±0.007301	0.145
Bilophila	0.000010±0.000049	0.000490±0.000918	<0.001
Shigella	0.006850±0.027674	0.051960±0.104650	0.002
